# Difference in Buccal Gingival Thickness between the Mandible and Maxilla in the Aesthetic Zone: A Systematic Review and Meta-Analysis

**DOI:** 10.3390/jcm13061789

**Published:** 2024-03-20

**Authors:** Linda Schwarz, Oleh Andrukhov, Marco Aoqi Rausch, Xiaohui Rausch-Fan, Erwin Jonke

**Affiliations:** 1 Division of Orthodontics, University Clinic of Dentistry, Medical University of Vienna, 1090 Vienna, Austria; linda.schwarz@meduniwien.ac.at (L.S.); marco.rausch@meduniwien.ac.at (M.A.R.); erwin.jonke@meduniwien.ac.at (E.J.); 2 Competence Center for Periodontal Research, University Clinic of Dentistry, Medical University of Vienna, 1090 Vienna, Austria; oleh.andrukhov@meduniwien.ac.at; 3 Center of Clinical Research and Department of Conservative Dentistry and Periodontology, University Clinic of Dentistry, Medical University of Vienna, 1090 Vienna, Austria

**Keywords:** aesthetic zone, gingival thickness, periodontal diagnostic, systematic review, transgingival probing, ultrasound measurement

## Abstract

**Background:** Fragile gingival tissue is a risk factor for the development of gingival recessions. Despite the fact that gingival recessions are more commonly seen around anterior mandibular teeth, previous research has predominantly concentrated on the gingival dimensions in the anterior maxilla. The objective was to systematically compare buccal gingival thicknesses between the upper and lower jaws in individuals with healthy gingival conditions in the aesthetic zone. **Methods:** A comprehensive search of three databases was carried out until October 2023. Gingival thickness differences between the maxilla and mandible were evaluated by calculating the mean differences along with the corresponding 95% confidence interval (CI). Subgroup analysis was conducted based on the measurement area, measurement method, and tooth category. **Results:** A total of seventeen studies were included in this systematic review. Eleven studies were included in the quantitative analysis. Quantitative analysis comparing gingival thickness around 2100 teeth in the anterior mandible to 2056 teeth in the anterior maxilla revealed a statistically significant thinner buccal gingiva in the mandible (mean difference: 0.16 mm; 95% CI [−0.24, −0.07]; *p* = 0.0003). **Conclusions:** The present systematic review revealed a more delicate buccal gingiva in the anterior mandible. However, further scientific validation is required due to the considerable heterogeneity in study design and the potential presence of confounding variables.

## 1. Introduction

The presence of gingival recessions is aesthetically unacceptable for many patients [1] and can lead to tooth hypersensitivity [2]. In addition, the denuded root surfaces are exposed to the oral environment and may be associated with carious lesions, abrasions, or erosions [3]. Various factors such as sex, age, anatomical location [4], tooth misalignment [5], occlusal trauma [6], and inflammation [7,8] have been suggested as potential contributors. However, the precise causal relationship between these factors and gingival thickness remains unclear.

Two main categories of gingiva morphology have been introduced: scalloped and thin, or flat and thick gingiva [9]. A thin phenotype is characterized by a narrower zone of attached tissue and a thinner facial–lingual gingival dimension [10,11,12]. Existing evidence suggests that individuals with thin and narrow gingiva tend to have more gingival recessions compared to those with thick and wide gingiva [13]. The presence of gingival recessions in the aesthetic zone may impact implant-supported prosthodontics after tooth extraction due to the resulting asymmetry of the implant bone level. To ensure optimal results in implant-supported prosthodontics after tooth extraction, the use of socket preservation techniques and subsequent soft tissue augmentation should be considered [14,15,16]. Gingival thickness exhibits significant variations both among individuals and within the same individual. The discrepancies in tissue thickness across different apico-coronal levels from the teeth and the lack of consensus on the appropriate anatomical reference point might explain the inconsistencies in earlier studies when defining gingival phenotypes [12,17,18].

Several quantitative measurement techniques have been proposed to analyze gingival thickness; accurate yet invasive methods involve the transgingival insertion of an endodontic instrument [19,20], a needle [11], or a periodontal probe [21]. Cone-beam computed tomography (CBCT) allows quantitative assessment of alveolar bone and gingiva [22], but at the expense of patient radiation exposure. On the other hand, ultrasonic devices [4] provide a non-invasive approach for measuring gingival dimensions. Other methods, albeit less common, include measurements via optical coherence tomography [23] or parallel radiographs with metal plates placed on soft tissues [24].

Lower incisors have been shown to be particularly susceptible teeth to developing labial recessions [25]. Even though the incidence of gingival recessions is highest in the anterior mandible, the majority of investigations examining gingival thickness predominantly focus on the upper jaw’s tissue dimensions [13]. This emphasis on the upper jaw might arise from the importance of tissue dimensions in achieving aesthetically satisfying outcomes with prosthetic restorations of upper incisors. A recent systematic review [22] examined the association between measurements obtained from CBCT and direct measurements via transgingival probing and could only identify one single study addressing the thickness of gingiva in lower incisors [26]. Clinical trials investigating both maxillary and mandibular tissue dimensions in the aesthetic zone (including the canines, lateral incisors, and central incisors) are rare; therefore, it remains unclear whether the susceptibility of lower incisors to gingival recessions can be attributed to more delicate gingival dimensions.

Consequently, the objective of this systematic review was to compare the dimensions of facial gingival dimensions in the aesthetic zone of the mandible with those in the maxilla. Moreover, the secondary objectives included evaluating potential influencing factors such as sex, age, measurement site, tooth classification, and measurement technique.

## 2. Materials and Methods

### 2.1. Inclusion and Exclusion Criteria

This review was carried out in accordance with the Preferred Reporting Items for Systematic Reviews and Meta-Analyses (PRISMA 2020) guidelines [27]. The construction of the research question was guided by the Population, Exposure, Comparison, and Outcome (PECO) framework, and it unfolded as follows:

P: Among the general population with a healthy periodontal status, what is the difference between;
E: buccal gingiva thickness in the aesthetic zone in the maxilla versus;
C: the mandible;
O: around anterior teeth (including canines, lateral incisors, and central incisors) in absolute numbers (millimeters).

This systematic review considered all study designs for inclusion, encompassing randomized controlled trials, cohort trials, case–control studies, and cross-sectional studies. All study designs were considered eligible for data synthesis, as gingival thickness measurements were mainly conducted in observational studies that did not require or justify any study interventions. There were no constraints imposed regarding the date of publication. Studies not presented in the English language or those in the form of editorial letters and reviews were excluded.

Individuals with permanent dentition and a healthy periodontal status were included. A healthy periodontal status was defined as the absence of horizontal or vertical bone loss, gingival recessions, periodontal pockets measuring ≥4 mm, calculus formation, and signs of gingival inflammation in the region of interest. Eligible measurement methods were transgingival probing, ultrasound, or radiographs. Trials that utilized a binary measurement method or solely reported on the width of the attached gingiva were excluded since they could not provide information about the level of gingival thickness.

Additionally, the following exclusion criteria were adopted: studies involving patients with systemic diseases, a history of smoking or the use of medications affecting periodontal tissues, as well as those with a history of soft tissue augmentation around anterior teeth, orthodontic treatment history, or dental restorations extending beyond the cementoenamel junction.

### 2.2. Search Strategy and Study Selection

The electronic literature search included the MEDLINE database (via PubMed), the Cochrane Library (via Ovid), and Embase from inception until 15 October 2023 (see Table S1 in the Supplementary Materials). In addition, the reference lists of relevant review articles, relevant excluded articles, and all included articles were screened. After the initial identification of records, the abstracts were imported into the software Endnote (Endnote X9 3.3, Frankfurt am Main, Germany) and automatically screened for duplicates, which were then removed. The initial reviewing process involved two researchers (L.S. and M.A.R.) independently evaluating the first 100 titles and abstracts. Any discrepancies that arose were discussed to achieve consensus. Next, the remaining records were divided equally between the reviewers, with each reviewer evaluating half of the records. If the title and abstract did not provide sufficient information to ascertain relevance, an examination of the full texts was conducted. A random subset of 100 records that were initially excluded by reviewer 1 was re-evaluated by reviewer 2, and conversely, a random subset of 100 records excluded by reviewer 2 was re-evaluated by reviewer 1. If there were disagreements regarding which full texts should be retrieved, a consensus was reached through discussion. Subsequently, both reviewers independently assessed the full texts for final inclusion. In instances where disagreements arose, a third reviewer (X.R.-F.) was consulted. The systematic review protocol was registered on the Open Science Framework under https://osf.io/pcv4h (accessed on 19 March 2024)

### 2.3. Data Collection Process and Data Items

The data from the included studies were gathered by one reviewer (L.S.) and subsequently cross-verified by another independent reviewer (O.A.). The following details were extracted from each of the studies included, utilizing a predetermined data extraction table: (1) general information, such as the first author, title, publication year, and journal title; (2) study characteristics, i.e., the study design and country in which it was conducted; (3) participant characteristics, comprising the sex and age of participants, along with their demographic status; (4) intervention characteristics, including the classification of teeth, location of measurement sites, the number of sites evaluated, and the evaluation methodology employed; and (5) outcomes of interest, i.e., the thickness of facial gingiva (measured in the oro-facial direction) at baseline—prior to the initiation of orthodontic treatment, augmentation procedures, or other interventions. The extracted measurements were taken on a per-tooth basis, per arch, or per designated area (differentiating between the anterior and posterior parts of the jaw).

### 2.4. Risk of Bias Assessment

All of the included studies were non-randomized trials with a cross-sectional observative design; therefore, the risk of bias was assessed using a tool for non-randomized studies (RoBANS [28]). RoBANS was harmonized with the Cochrane’s RoB tool and GRADE (Grading of Recommendations Assessment, Development, and Evaluation) [29]. RoBANS addresses the following six domains: (1) the selection of participants; (2) confounding variables; (3) measurement of exposure; (4) blinding of outcome assessments; (5) incomplete outcome data; and (6) selective outcome reporting [28]. The assessment was conducted by one reviewer (L.S.) and checked by another (O.A.). To assess the risk of bias with the RoBANS tool, the following major confounding variables were specified: the sex and age of the participants and a history of orthodontic treatment.

### 2.5. Method of Data Synthesis

In order to conduct a systematic quantitative data synthesis, the eligible studies were organized and grouped based on several critical dimensions. These dimensions were selected to ensure the comparability of these studies and facilitate a comprehensive analysis of tissue thickness variations between the upper and lower jaws. The following categorization criteria were applied:

1. Mid-buccal vs. interdental measurements: Studies were classified based on whether tissue thickness was measured along the long axis of the tooth at mid-buccal sites or interdentally between the teeth.
2. Apico-coronal measurement area: The location of the tissue thickness measurement along the vertical dimension was considered to account for potential variations at different apico-coronal levels. Due to the inconsistent use of landmarks amongst the studies, two main categories were defined by the authors: (1) supracrestal measurements, including measurements at the base of the sulcus or of the papilla and at the cementoenamel junction (CEJ); and (2) subcrestal measurements, including measurements in the attached gingiva or measurements halfway between the gingival crest and mucogingival junction.
3. Measurement method: The studies were organized according to the measurement method that was employed for gingiva thickness assessment, encompassing transgingival measurements using a probe or a needle to penetrate the soft tissue, ultrasound measurements, or radiographic examinations.
4. Tooth classification: Finally, the classification of teeth (incisors vs. canines) was extracted to account for potential variations in gingiva thickness between different tooth types.

For inclusion in the quantitative data analysis, only studies with comparable measurement sites were considered. This approach aimed to ensure that the data synthesized pertained to similar anatomical locations within the oral cavity. Only studies reporting on mean values with standard deviations were considered for our data analysis. In instances where a study reported the standard error or the mean, the standard deviation was computed to ensure uniformity in the data. Only studies published in journals indexed by Clarivate’s Web of Science were considered for data extraction to ensure a sufficient scientific reliability despite the observational study character. Whenever data were accessible on a per-tooth basis, this information was used to compute the mean difference and associated confidence interval across all of the studies. In cases where the included study did not provide data on gingiva thickness per tooth, the reported mean value across all anterior teeth, ranging from canine to canine, was used for our quantitative analysis. Due to the limited number of identified studies providing separate data for both genders or different age groups, it was not feasible to quantitatively examine the impact of age and gender on gingiva thickness. The following subgroups were defined for quantitative data analysis: (1) supracrestal vs. subcrestal measurements; (2) ultrasound vs. transgingival measurements; and (3) thickness around the incisors vs. canines.

A random effects (REs) meta-analysis, together with the inverse variance-weighted average method, was chosen to account for individual study effects. The statistical heterogeneity was calculated using the Cochran’s Q and the I_2_ Index, which describes the percentage of variation in the global estimate. Statistical analyses were conducted with RevMan Web software (The Cochrane Collaboration, https://revman.cochrane.org, accessed on 19 March 2024).

## 3. Results

### 3.1. Study Selection and Characteristics

**Figure 1:**
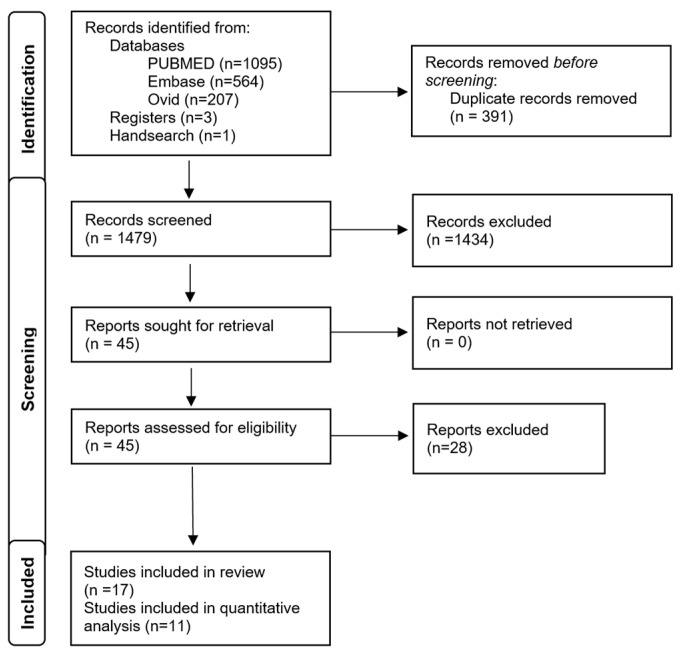
PRISMA flow diagram for the identification of relevant studies.

A flowchart representing the search approach in accordance with the “Preferred Reporting Items for Systematic reviews and Meta-Analyses (PRISMA) statement” [27] is displayed in Figure 1. The combination of electronic and manual searches yielded a total of 1870 records, out of which 391 duplicates were eliminated. The titles and abstracts of the remaining articles were evaluated, resulting in the exclusion of 1434 records. This led to a total of 45 full-text versions being retrieved. After the exclusion of 28 reports, a total of 17 studies were included into this systematic review (see the Supplementary Materials for exclusion reasons).

All of the included studies were observational and had been published within a timeframe ranging from 1971 [30] to 2022 [31,32,33]. The design of all of the studies was cross-sectional. The number of participants ranged from 8 [30] to 128 [34]. For the measurement of gingiva thickness, twelve studies employed transgingival probing, five studies utilized ultrasound technology, and one study used CBCT datasets (see Table 1). When it came to transgingival measurements, the most common tools were endodontic needles or spreaders with sharp tips. However, three studies made use of periodontal probes with blunt tips [21,35,36]. Ultrasound measurements were conducted with a SDM ultrasonic gingival thickness meter (Krupp Corporation, Essen, Germany) [4,37,38], the HD11 Ultrasound system (Philips Healthcare, Hamburg, Germany) [39], and a device with no further specification [30].

Two studies did not provide any information regarding the number of male and female participants [30,34] (Table 2). Six studies [21,26,31,36,38,41] conducted separate data analysis for both genders. Eger et al. included only male participants [4]. In the study by Lee et al. [42], data for men and women were not reported separately, but it was mentioned that there was no significant difference between the sexes. Among included studies, six of them provided the patients’ mean age and thirteen the patients’ age range (see Table 2). Five studies examined different age groups [4,21,36,41,42].

Most of the studies, specifically 12 out of 17, focused on mid-buccal measurements (Table 3). Most measurements were conducted mid-buccally along the long axis of the tooth [4,21,26,31,32,34,35,36,37,39,40,41,42,43,44]. Alternatively, measurements of gingival thickness were taken between the teeth, i.e., in interdental areas [21,31,36,38]. A single study exclusively reported interdental measurements [38]. Three studies reported on the mean values of two different measurement points [34,43,44], and three studies conducted measurements on two or more different measurement sites and reported their corresponding values [32,39,40].

### 3.2. Differences in Gingiva Thickness between the Maxilla and Mandible

Four studies [21,31,36,41] indicated a greater gingival thickness in the anterior mandible when compared to the maxilla. Conversely, all other studies included in this review suggested thicker gingiva in the anterior maxilla (see Table 3). At the level of the CEJ, Shao et al. [26] observed that the mean gingival thickness was 1.21 ± 0.27 mm in the anterior maxilla and 0.85 ± 0.24 mm in the anterior mandible (*p* < 0.005), and Han et al. [32] reported 1.29 ± 0.33 mm in the anterior maxilla and 0.93 ± 0.21 mm in the anterior mandible. At the base of the sulcus, Eger et al. reported 1.28 ± 0.40 mm around the upper central incisors and 0.87 ± 0.33 mm around the lower central incisors measured by ultrasound technology [4]. Similarly, Müller et al. measured 1.00 ± 0.30 mm and 0.65 ± 0.14 mm, respectively [37]. As for the measurements conducted by transgingival probing, the gingival thickness was 1.23 mm [40] and 1.3 mm [31] around the upper central incisors and 0.89 mm [40] and 1.6 mm [31] around the lower central incisors at the base of the sulcus. In the study conducted by Anand et al. [31], interdental measurements showed higher values in the maxilla, while mid-buccal measurements yielded higher values in the mandible. Gingival thickness at the landmark “midway between gingival crest and mucogingival junction” was 1.28 mm [40] and 1.55 mm [42] around the central incisors in the maxilla, vs. 0.89 mm [40] and 1.01 [42] mm in the mandible. Three studies [37,42,43] reported consistently thinner gingiva in the mandible compared to maxillary measurements; however, they did not provide statistical analyses. Similarly, other studies reported mean values for maxillary and mandibular gingival thickness (see Table 3), but did not statistically interpret the results or address differences between the arches [4,30,32,35,40,44].

### 3.3. Data Synthesis

Eleven studies were considered eligible for quantitative data analysis [4,21,26,30,31,32,35,36,37,40,42]. These studies were selected because they offered results that could be compared effectively, as their common focus was the measurement of gingival thickness around the anterior teeth, with the measurements being taken mid-buccally along the longitudinal axis of the respective tooth. One study was excluded from quantitative data synthesis as it solely reported on interdental measurements [38]. Two other studies were not considered eligible for quantitative data analysis as the published data did not describe clearly assignable measurement points but rather presented averages from different measurement locations [34,44]. Three studies were published in scientific journals not indexed in Clarivate’s Web of Science and were therefore not included in our quantitative data analysis [39,41,43]. Four studies had comparable locations of measurement sites in the supracrestal region and five studies in the subcrestal region. Two studies [32,40] provided information on both regions. One included study reported on the standard error of the mean; therefore, the standard deviation had to be calculated [30].

#### 3.3.1. Risk of Bias within Studies

Four studies [4,21,32,36] were of unclear risk of selection bias due to the absence of a reported recruitment timeframe (see Table 4 and Table S2 in the Supplementary Materials). One study was categorized as posing a high risk of selection bias since it did not provide any inclusion or exclusion criteria [30]. Additionally, 2 out of 11 studies (18.2%) were deemed to carry a high risk of selection bias because they did not adequately identify or adjust for more than one major confounding variable [30,40]. Five out of eleven studies (45.4%) did not identify a single major confounding variable and were therefore classified as having an unclear risk of bias [4,21,32,35,36]. The main concern of the included studies was a selection bias, which affected 63% of all studies. One study was classified as presenting a high risk of performance bias due to the use of experimental measurement techniques [30]. No study was found to have a risk of attrition bias or reporting bias. Justifications for assessments of bias for individual studies are available in the Supplementary Materials.

#### 3.3.2. Results of Synthesis: Difference in Gingival Thickness between the Upper and Lower Jaws

**Figure 2:**
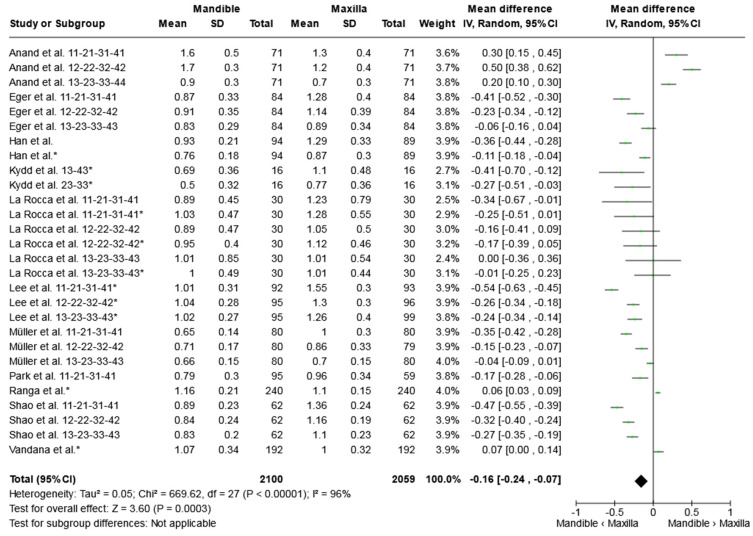
Meta-analysis of studies comparing gingival thickness around the anterior teeth between the mandible and maxilla. * Asterix indicates the values of measurements in the subcrestal region. The mean difference of gingiva thickness between the upper and lower jaw is represented as a green dot along a horizontal line, denoting the confidence interval of each study. The collective result from all studies is illustrated by a black diamond, indicating the aggregated effect size. References: Anand et al. 2022 [31], Eger et al. 1996 [4], Han et al. [32], Kydd et al. [30], La Rocca et al. 2012 [40], Lee et al. 2017 [42], Müller et al. 2000 [37], Park et al. 2018 [35], Ranga et al. 2015 [36], Shao et al. 2018 [26], Vandana et al. 2005 [21].

A REs meta-analysis of 11 studies was performed, and the pooled mean difference in gingival thickness between the upper and lower jaws in the aesthetic region was assessed (Figure 2). Gingival thickness around a total of 2100 teeth in the mandible was compared with measurements around 2059 teeth in the maxilla. According to the present meta-analysis, there is a statistically significant difference between the gingival thickness between the upper and lower jaws. The facial gingiva in the aesthetic zone was seen to be 0.16 mm (95% CI: −0.24, −0.07) thinner in the mandible compared to the maxilla (*p* = 0.0003). The chi-square test for heterogeneity was significant (*p* < 0.001).

#### 3.3.3. Subgroup Analysis

Subgroup analysis suggests that there are no statistically significant differences between the subgroups (see Figure S1 in the Supplementary Materials). The mean difference between the upper and lower jaws was 0.19 mm for the subcrestal subgroup and 0.14 mm for the supracrestal subgroup (*p* < 0.001). However, the I^2^ value, which measures study heterogeneity within each subgroup, remained high at 96% (Table 5). The most pronounced difference between the upper and lower jaws was observed in the subgroups of ultrasound and CBCT measurements, with 0.23 mm less gingiva thickness in the mandible compared to the maxilla. Neither the analysis of the subgroups “measurement method”, “tooth category”, nor “measurement area” (see Figure S1) detected any statistically significant subgroup differences (*p* = 0.40, *p* = 0.43, and *p* = 0.60, respectively).

#### 3.3.4. Risk of Bias across Studies

To rate the risk of bias across studies, the GRADE approach was adopted (see Table S3 in the Supplementary Materials). As all of the included studies were observational studies, the body of evidence was considered to start as low-quality evidence. As 63% of the included studies did not adequately control for confounding factors, the body of evidence was rated down one level to very low quality.

## 4. Discussion

This systematic review and meta-analysis have demonstrated that (1) a statistically significant difference between maxillary and mandibular gingiva thickness in the aesthetic zone could be detected, and (2) existing evidence on the difference between maxillary and mandibular gingival thickness is limited and mostly consists of cross-sectional observational studies. Although the overall body of evidence was deemed to be of very low quality, the consistently thinner gingival thickness in the mandible may contribute to a higher incidence of gingival recessions compared to the maxilla.

The large heterogeneity of results observed across the included studies may be attributed to differences in the characteristics of the study populations. Subgroup analysis could not reduce the heterogeneity of the study results included in the quantitative analysis. Due to the limited number of studies considering gender and age differences, it was not feasible to conduct separate analyses for subpopulations based on age and gender. The patients exhibited a wide age range, spanning from 16 to 49 years. Young adults (aged 16 to 24 years) exhibited significantly thicker gingiva in the anterior mandibular region compared to the older age group [21,36]. Specifically, transgingival measurements showed an average gingiva thickness of 1.22 ± 0.29 mm [36] and of 1.73 ± 0.37 mm [21] for young adults. In contrast, the older age group (25 to 38 years) had significantly smaller gingiva dimensions, with an average thickness of 1.04 ± 0.11 mm [36] and 1.03 ± 0.31 mm [21]. However, two studies conducted by Lee et al. [42] and Eger et al. [4] did not find any age-dependent differences in gingiva dimensions. Although it can be assumed that differences between the upper and lower jaws persist as individuals age, there is a lack of scientific data supporting this assumption.

Several studies detected consistent differences between the upper and lower jaws among different genders, as the gingiva was found to be thinner in females compared to males [21,26,31,36,38]. However, in two studies, these differences were not statistically significant [38,44]. In the study conducted by Anand et al. [31], it was observed that women exhibited significantly thinner gingiva, with an average thickness of 1.2 ± 0.1 mm across all teeth, as opposed to men, who had a thickness of 1.4 ± 0.1 mm (*p* < 0.001). Accordingly, three studies reported a significantly thinner gingiva in females in the anterior mandibula: At the halfway distance between the mucogingival junction, gingiva thickness measured 1.27 ± 0.23 mm in men and 1.02 ± 0.07 mm in women (*p* < 0.001) [36]. At the base of the papilla, Vandana et al. [21] reported values of 1.22 ± 0.38 mm in men and 1.06 ± 0.42 mm in women (*p* < 0.02). At the cementoenamel junction, gingiva thickness was found to be 1.06 ± 0.32 mm in men and 0.99 ± 0.30 mm in women (*p* < 0.005) [26]. Lastly, the study conducted by Parmar et al. noted significant differences between the canines and first premolars, but not between other teeth [45]. However, the effect of gender on gingival recession remains inconclusive [46,47].

The gingival phenotype varies within and between individuals, and the role of genetically determined ethnicity on the thickness of the gingiva has already been suggested [13]. In the present review, gingiva thickness was reported to be consistently higher in the maxilla, with the exception of three studies [21,31,36]. In contrast to the other studies, these three studies reported on greater gingival dimensions in the mandible. They were all conducted in India, suggesting potential ethnic variations in gingival dimensions within this population. The findings of the present review suggest a significant variation in tissue dimensions among different populations, which highlights the importance of soft tissue augmentation procedures for certain clinical implications. In clinical situations where there is insufficient soft tissue, mucosal grafts are often used in periodontal and implant dentistry to cover gingival recessions [48], to reconstruct lost dental papillae [49], or to establish a healthy peri-implant environment [50]. Autogenous grafts, such as free gingival grafts and subepithelial connective tissue grafts, are widely considered the gold standard due to their superior biocompatibility and integration with host tissues [51]. While allografts and xenografts eliminate the need for a donor site [52], they may not offer the same level of biocompatibility and integration as autogenous grafts. However, growth factors and biologically active molecules, such as enamel matrix derivatives and platelet-rich fibrin (PRF), are increasingly used to enhance wound healing and tissue regeneration [53]. The latest generations of PRF offer distinct benefits for tissue regeneration and healing. For example, T-PRF (titanium-prepared PRF) is a more advanced and aggregated form of fibrin in comparison to PRF [50], while L-PRF (leukocyte- and platelet-rich fibrin) is characterized by the inclusion of a significant number of leukocytes [54].

The measurement method may affect the evaluation of tissue dimensions. Transgingival measurements, as well as ultrasound and radiographic evaluations, do not represent the true tissue dimensions but rather an approximation thereof. Of the studies included in this review, three used blunt instruments for transgingival measurements of gingival dimensions [21,35,36]. This may be another source of heterogeneity, as different instruments for transgingival measurements may lead to different results. A previous study by Kloukos et. al, comparing measurements obtained with blunt (periodontal probe) and sharp (acupuncture needle) instruments, found differences in measurements depending on the thickness of the gingiva [55]. Specifically, for every 1 mm increase in gingival thickness, the measurements obtained with a sharp instrument were expected to be 22% greater than those obtained with a blunt instrument. This highlights the importance of the instruments used for measurement when interpreting and comparing results between studies. Subgroup analysis could not confirm any systematic deviation in gingival thickness difference between the different measurement groups (i.e., transgingival probing, ultrasound, and CBCT). This, however, does not exclude deviations from the measured values between the groups but rather confirms that the differences between the maxilla and mandible remain constant within the measurement method.

It is noteworthy that there is no uniform nomenclature for the diverse zones or reference points of the gingiva. The literature employs various terminologies, some of which are overlapping, thereby confounding the interpretation of outcomes. For example, common landmark definitions used in literature are “marginal gingiva”, which corresponds to “free gingiva”. Consequently, “free gingival margin” is used synonymously with “crest of marginal gingiva” [34,35,37]. The free gingiva forms the soft tissue wall of the gingival sulcus and can be separated from the tooth surface by periodontal probing [56]. However, La Rocca et. al. [40] chose the measurement points to be “1 mm apical to the facial probing depth” and designated this landmark as “gingival margin”. In other studies, “marginal gingiva” was considered equivalent to “1 mm apical to marginal groove” [39]. Finally, the thickness of the gingiva at the base of the sulcus was referred to as “free gingiva thickness” [57]. It is therefore necessary to study the scientific papers carefully in order to be able to interpret the results appropriately.

The available literature proposes various measurement locations. The measurement sites that were used by the studies included in the present review differed with regard to apico-coronal levels and the location of dedicated reference points. Defined measurement sites were “1 mm apical to the pocket base” [35], “a point corresponding to the base of the sulcus”/“1 mm apical from probing depth” [4,21,31,37,40], “midway between sulcus and mucogingival junction [21,40,42], and “as apical as possible in the attached gingiva”/“1 mm coronal from mucogingival junction” [38,40]. The literature reporting on facial gingiva thickness that was not included in the present review reports on even more different measurement locations. They are situated either 1 mm [1], 1.5 mm [58], 2 mm [59], or 3 mm [60] from the gingival margin or at a distance of 3 mm from the cementoenamel junction [61]. This makes it difficult to directly compare the results of different studies.

In order to facilitate meaningful comparisons of gingival measurements between studies, the adoption of a standardized methodology for the assessment of gingival dimensions would be highly beneficial. In addition, there is a need for further research efforts in this area that systematically control for patient age and gender. By conducting comprehensive studies in this regard, it will be possible to gain more robust evidence on the influence of these factors on gingival dimensions and to reduce the heterogeneity of existing data.

## 5. Conclusions

Based on the data found in this systematic review and meta-analysis, the following conclusions can be drawn: (1) the mean gingival thickness in the aesthetic zone is slightly lower (0.16 mm) in the lower jaw; (2) different measurement methods yield consistent results when comparing gingival thickness between the maxilla and mandible. However, different measurement techniques introduce a risk of bias when comparing absolute gingival thickness between studies. (3) There is a need for further standardized research considering possible confounding factors such as sex, age, and history of orthodontic treatment.

## Figures and Tables

**Table 1:** Main characteristics of included studies.

First Author and Year	Country	Setting	Study Design	Measurement Method
Kydd et al. (1971) [30]	United States	University	Prospective cross-sectional	Ultrasonic
Eger et al. (1996) [4]	Germany	German Armed Forces Central Hospital	Prospective cohort study	Ultrasonic
Müller et al. (2000) [37]	Germany	German Armed Forces Central Hospital	Prospective cross-sectional	Ultrasonic
Vandana et al. (2005) [21]	India	University	Cross-sectional	Transgingival probing
Cha et al. (2008) [38]	Republic of Korea	University	Prospective cross-sectional	Ultrasonic
La Rocca et al. (2012) [40]	Spain	University	Prospective cross-sectional	Transgingival probing
Sharma et al. (2014) [39]	India	University	Cross-sectional	Transgingival probing + ultrasonic
Kolte et al. (2014) [41]	India	University	Prospective cross-sectional	Transgingival probing
Ranga et al. (2015) [36]	India	University	Cross-sectional	Transgingival probing
Lee et al. (2017) [42]	Malaysia	Private	Prospective cohort study	Transgingival probing
Kaya et al. (2018) [34]	Turkey	University	Prospective cross-sectional	Transgingival probing
Park et al. (2018) [35]	Republic of Korea	University	Prospective cross-sectional	Transgingival probing
Shao et al. (2018) [26]	China	University	Prospective cross-sectional	Transgingival probing + CBCT
Rathod et al. (2020) [43]	India	University	Prospective cross-sectional	Transgingival probing
Alkan et al. (2021) [44]	Turkey	University	Before-after study	Transgingival probing
Anand et al. (2022) [31]	India	University	Prospective cross-sectional	Transgingival probing
Han et al. (2022) [32]	China	University	Cross-sectional	CBCT + intraoral scan

Abbreviations: CBCT, cone-beam computed tomography.

**Table 2:** Sample characteristics of included studies.

	Sample Characteristics					
First Author and Year	Sample Size Calculation	Study Population	Patients (N)	Sex (Female)	Age (Years)	
					Mean (±SD)	Range
Kydd et al. (1971) [30]	N	NR	8	NR	NR	24–41
Eger et al. (1996) [4]	N	Healthy volunteers from the Armed Forces Central Hospital	42	0	NR	20–25
Müller et al. (2000) [37]	N	Dental staff	40	21	NR	19–30
Vandana et al. (2005) [21]	N	Systemically healthy subjects	32	16	NR	16–38
Cha et al. (2008) [38]	N	Young adults	61	33	25.3	19–35
La Rocca et al. (2012) [40]	N	Subjects in need for orthodontic treatment or implant placement	15	7	29.53	22–49
Sharma et al. (2014) [39]	N	Subjects visiting the Department of Periodontics	30	15	NR	18–30
Kolte et al. (2014) [41]	Y	Systemically healthy adults	120	60	NR	Three age groups: 16–24, 25–40, and >40
Ranga et al. (2015) [36]	N	Systemically healthy subjects from Department of Periodontics	40	20	NR	16–38
Lee et al. (2017) [42]	Y	Subjects from National Dental Center	51	27	30.4 ± 11.4	NR
Kaya et al. (2018) [34]	Y	Subjects from orthodontic department	128	NR	16.79 ± 3.66	NR
Park et al. (2018) [35]	Y	Patients with skeletal class III malocclusion	28	9	21.15 ± 4.02	NR
Shao et al. (2018) [26]	N	Students	31	16	NR	18–30
Rathod et al. (2020) [43]	Y	Patients at Department of Periodontics and Implantology	110	53	NR	18–30
Alkan et al. (2021) [44]	N	Subjects in need of fixed orthodontic treatment	40	20	16.65 ± 3.23	NR
Anand et al. (2022) [31]	Y	Students or staff	71	39	NR	19–30
Han et al. (2022) [32]	N	Patient with skeletal class III malocclusion and thin alveolar bone	24	15	NR	18–30

Abbreviations: NR, not reported; N, no; and Y, yes.

**Table 3:** Intervention and outcome characteristics of included studies.

	Intervention Characteristics	Outcome Characteristics			
Study	Measurement Site	Measurement Area	Measurement Method	Gingiva Thickness (mm)	
				Maxilla (Tooth) ^1^	Mandible (Tooth) ^1^
Kydd et al. (1971) [30] ^2^	Attached gingiva	Mid-buccally	Ultrasound	1.1 ± 0.48 (13)	0.69 ± 0.36 (43)
				0.77 ± 0.36 (23)	0.50 ± 0.32 (33)
Eger et al. (1996) [4]	Base of sulcus	Mid-buccally	Ultrasound	1.28 ± 0.40 (11, 21)	0.87 ± 0.33 (31, 41)
				1.14 ± 0.39 (12, 22)	0.91 ± 0.35 (32, 42)
				0.89 ± 0.34 (13, 23)	0.83 ± 0.29 (33, 43)
Müller et al. (2000) [37]	Base of sulcus	Mid-buccally	Ultrasound	1.00 ± 0.30 (11, 21)	0.65 ± 0.14 (31, 41)
				0.86 ± 0.33 (12, 22)	0.71 ± 0.17 (32, 42)
				0.70 ± 0.15 (13, 23)	0.66 ± 0.15 (33, 43)
	Base of papilla	Interdental	Ultrasound	1.86 ± 0.45 (11, 21)	1.13 ± 0.64 (31, 41)
				1.32 ± 0.38 (12, 22)	1.02 ± 0.42 (32, 42)
				1.34 ± 0.45 (13, 23)	1.43 ± 0.57 (33, 43)
Vandana et al. (2005) [21]	Midway	Mid-buccally	Transgingival	1.00 ± 0.32	1.07 ± 0.34
				(13, 12, 11, 21, 22, 23)	(43, 42, 41, 31, 32, 33)
	Base of papilla	Interdental	Transgingival	0.95 ± 0.35	1.13 ± 0.41
				(13, 12, 11, 21, 22, 23)	(43, 42, 41, 31, 32, 33)
Cha et al. (2008) [38]	Attached Gingiva	Interdental	Ultrasound	Men:	Men:
				1.20 ± 0.18 (11/21)	1/1: 1.07 ± 0.26 (31/41)
				1.84 ± 0.53 (11/12, 21/22)	1/2: 1.07 ± 0.18 (41/42, 31/32)
				1.48 ± 0.44 (12/13, 22/23)	2/3: 1.27 ± 0.40 (42/43, 32/33)
				Women:	Women:
				1.28 ± 0.17 (11/21)	1/1: 1.09 ± 0.29 (31/41)
				1.48 ± 0.48 (11/12, 21/22)	1/2: 1.18 ± 0.33 (41/42, 31/32)
				1.26 ± 0.31 (12/13, 22/23)	2/3: 1.24 ± 0.36 (42/43, 32/33)
La Rocca et al. (2012) [40]	Base of sulcus	Mid-buccally	Transgingival	1.23 ± 0.79 (11, 21)	0.89 ± 0.45 (31, 41)
				1.05 ± 0.50 (12, 22)	0.89 ± 0.47 (32, 42)
				1.01 ± 0.54 (13, 23)	1.01 ± 0.85 (33, 43)
	Midway	Mid-buccally	Transgingival	1.28 ± 0.55 (11, 21)	1.03 ± 0.47 (31, 41)
				1.12 ± 0.46 (12, 22)	0.95 ± 0.40 (32, 42)
				1.01 ± 0.44 (13, 23)	1.0 ± 0.49 (33, 43)
Sharma et al. (2014) [39]	Marginal groove	Mid-buccally	Transgingival	0.587 ± 0.08 (12)	0.574 ± 0.05 (42)
				0.574 ± 0.06 (22)	0.556 ± 0.07 (32)
			Ultrasound	0.578 ± 0.08 (12)	0.564 ± 0.05 (42)
				0.566 ± 0.06 (22)	0.557 ± 0.07 (32)
	Mucogingival junction	Mid-buccally	Transgingival	0.961 ± 0.07 (12)	0.926 ± 0.05 (42)
				0.924 ± 0.06 (22)	0.937 ± 0.04 (32)
			Ultrasound	0.951 ± 0.07 (12)	0.9279 ± 0.04 (42)
				0.925 ± 0.07 (22)	0.929 ± 0.04 (32)
Kolte et al. (2014) [41]	Midway	Mid-buccally	Transgingival	1.04 ± 0.52 (13, 12, 11, 21, 22, 23)	1.12 ± 0.69 (43, 42, 41, 31, 32, 33)
Ranga et al. (2015) [36]	Base of papilla	Interdental	Transgingival	1.30 ± 0.30 (13, 12, 11, 21, 22, 23)	1.44 ± 0.44 (43, 42, 41, 31, 32, 33)
	Midway	Mid-buccally	Transgingival	1.10 ± 0.15 (13, 12, 11, 21, 22, 23)	1.16 ± 0.21 (43, 42, 41, 31, 32, 33)
Lee et al. (2017) [42]	Midway	Mid-buccally	Transgingival	1.55 ± 0.30 (11, 21)	1.01 ± 0.31 (31, 41)
				1.30 ± 0.30 (12, 22)	1.04 ± 0.28 (32, 42)
				1.26 ± 0.40 (13, 23)	1.02 ± 0.27 (33, 43)
Kaya et al. (2018) [34]	Mean of two measurement sites	Mid-buccally	Transgingival	1.13 ± 0.23 (13, 12, 11, 21, 22, 23)	0.71 ± 0.16 (43, 42, 41, 31, 32, 33)
Park et al. (2018) [35]	1 mm apical to pocket base	Mid-buccally	Transgingival	0.96 ± 0.34 (11, 21)	0.79 ± 0.30 (31, 41)
Shao et al. (2018) [26]	CEJ	Mid-buccally	Transgingival	1.36 ± 0.24 (11, 21)	0.89 ± 0.23 (31, 41)
				1.16 ± 0.19 (12, 22)	0.84 ± 0.24 (32, 42)
				1.10 ± 0.23 (13, 23)	0.83 ± 0.20 (33, 43)
Rathod et al. (2020) [43]	Mean of two measurement sites	Mid-buccally	Transgingival	1.02 ± 0.26 (11)	0.80 ± 0.27 (31)
				1.00 ± 0.22 (21)	0.83 ± 0.28 (41)
				1.01 ± 0.25 (12)	0.83 ± 0.27 (32)
				1.00 ± 0.27 (22)	0.85 ± 0.27 (42)
				0.90 ± 0.26 (13)	0.76 ± 0.31 (33)
				0.93 ± 0.27 (23)	0.80 ± 0.31 (43)
Alkan et al. (2021) [44]	Mean of two measurement sites	Mid-buccally	Transgingival	1.24 ± 0.34 (11)	0.67 ± 0.17 (31)
				1.16 ± 0.45 (12)	0.76 ± 0.17 (32)
				0.82 ± 0.24 (13)	0.61 ± 0.19 (33)
				1.23 ± 0.29 (21)	0.70 ± 0.19 (41)
				1.20 ± 0.42 (22)	0.80 ± 0.28 (42)
				0.82 ± 0.27 (23)	0.61 ± 0.21 (43)
Anand et al. (2022) [31]	Base of sulcus	Mid-buccally	Transgingival	1.3 ± 0.4 (11, 21)	1.6 ± 0.5 (41, 31)
				1.2 ± 0.4 (12, 22)	1.7 ± 0.3 (42, 32)
				0.7 ± 0.3 (13, 23)	0.9 ± 0.3 (43, 33)
Han et al. (2022) [32]	CEJ	Mid-buccally	CBCT	1.29 ± 0.33 (13, 12, 11, 21, 22, 23)	0.93 ± 0.21 (43, 42, 41, 31, 32, 33)
	3 mm below CEJ	Mid-buccally	CBCT	0.87 ± 0.30 (13, 12, 11, 21, 22, 23)	0.76 ± 0.18 (43, 42, 41, 31, 32, 33)

Values are depicted as the mean ± standard deviation. Numbers in braces indicate the measurements’ corresponding teeth. Abbreviations: CEJ, cementoenamel junction; and CBCT, cone-beam computed tomography. ^1^ tooth numbering by FDI World Dental Federation notation. ^2^ standard deviation calculated from the standard error of the mean.

**Table 4:** Assessment of risk of bias according to RoBANS (Risk of Bias Assessment of Non-Randomized Studies).

	Selection Bias	Selection Bias	Performance Bias	Detection Bias	Attrition Bias	Reporting Bias
Risk of Bias	Selection of Participants	Confounding Variable	Measurement of Exposure	Blinding of Outcome Assessments	Incomplete Outcome Data	Selective Outcome Reporting
Low	6 (54.5%)	4 (36.4%)	10 (90.9%)	11 (100%)	11 (100%)	11 (100%)
Unclear	4 (36.4%)	5 (45.4%)	0 (0%)	0 (0%)	0 (0%)	0 (0%)
High	1 (9.1%)	2 (18.2%)	1 (9.1%)	0 (0%)	0 (0%)	0 (0%)

**Table 5:** Summary of findings.

**Difference of Buccal Gingival Thickness between the Lower and Upper Jaws**										
Population: Individuals with permanent dentition and healthy periodontal status. Setting: University or private practise. Intervention: Transgingival probing and ultrasound in the mid-buccal area. Comparison: Maxilla.										
**Outcomes**	**Subgroups**	**Categories**	**n Study**	**n Teeth**		**ME**	**95% CI**	***p*-Value**	**I^2^**	***p*-Value**
				**Mand**	**Max**					
Difference between the maxilla and mandible	Overall		10	2100	2059	−0.16	−0.24, −0.07	0.0003 *	96%	<0.001
	Measurement area	Subcrestal	6	836	842	−0.19	−0.32, −0.05	0.007 *	96%	<0.001
		Supracrestal	7	1170	1128	−0.14	−0.24, 0.00	0.02 *	96%	<0.001
	Measurement method	Transgingival	7	1388	1358	−0.11	−0.24, 0.01	0.08	97%	<0.001
		Ultrasound	3	524	523	−0.23	−0.34, −0.11	0.001 *	91%	<0.001
		CBCT	1	188	178	−0.23	−0.48, 0.01	0.06	95%	<0.001
	Tooth category	Incisors	7	1026	991	−0.19	−0.32, −0.06	0.003 *	95%	<0.001
		Canines	7	454	458	−0.12	−0.24, 0.00	0.05	91%	<0.001

The figure displays for each study included in the meta-analysis the summary statistics (mean, standard deviation, and sample size) of both groups (mandible and maxilla), the mean difference, and its 95% interval for the continuous outcome (that is gingiva thickness (mm)). * Asterix indicates statistically significant *p*-values at the level of less than 0.05%. Abbreviations: CI, confidence interval; I^2^, heterogeneity; ME, mean effect; Mand, mandible; and Max, maxilla.

## Data Availability

The data are available upon reasonable request from the authors.

## Supplementary Materials

The following supporting information can be downloaded at: https://www.mdpi.com/article/10.3390/jcm13061789/s1, Figure S1: Results of subgroup analysis; Table S1: Search strategy; Table S2: Risk of Bias; Table S3: Summary of findings using the Grading of Recommendations Assessment, Development and Evaluation (GRADE) approach. References [62,63,64,65,66,67,68,69,70,71,72,73,74,75,76,77,78,79,80,81,82,83,84,85] are cited in Supplementary Materials.
