# Supplementary material for: Difference in Buccal Gingival Thickness between the Mandible and Maxilla in the Aesthetic Zone: A Systematic Review and Meta-Analysis
Source: J Clin Med. 2024 Mar 20;13(6):1789. doi: 10.3390/jcm13061789 (PMC10971348; doi:10.3390/jcm13061789)
Supplement: Supplementary file 1 [file jcm-13-01789-s001.zip › jcm-2891398-supplementary.pdf]

Supplementary Data

Table S1: Search strategy

Database	Search strategy
MEDLINE (via PubMed) www.ncbi.nlm.nih.gov/pubmed/	((mandible) OR (lower jaw)) AND ((alveolar mucosa) OR (gingiva) OR (soft tissue) OR (mucosal)) AND ((thickness) OR (dimension))
Embase https://www.embase.com/	('thickness soft tissue' OR 'thickness'/exp OR thickness) AND ('soft tissue' OR 'mucosa'/exp OR 'gingiva'/exp) AND ('mandible'/exp OR mandible)
Clinical Trials, NIH* https://clinicaltrials.gov	Search term: gingival thickness or mucosa thickness WITH results: no studies found

Reasons for exclusion of scientific reports:

1) No measurement of gingival thickness (N=9)
    a. J.R. Perez, H. Smukler, M.E. Nunn, Clinical dimensions of the supraosseous gingivae in healthy periodontium, J Periodontol 79(12) (2008) 2267-72.
    b. H.C. Lim, J. Lee, D.Y. Kang, I.W. Cho, H.S. Shin, J.C. Park, Digital Assessment of Gingival Dimensions of Healthy Periodontium, J Clin Med 10(8) (2021).
    c. S. Kothiwale, J. Ajbani, Innovative use of increasing the width of attached gingiva using chorion membrane along with platelet rich fibrin membrane, Cell Tissue Bank 22(3) (2021) 389-398.
    d. J. Jiao, W.D. Jing, J.X. Hou, X.T. Li, X.X. Wang, X. Xu, M.X. Mao, L. Xu, Nomogram prediction of vulnerable periodontal condition before orthodontic treatment in the anterior teeth of Chinese patients with skeletal Class III malocclusion, Acta Odontol Scand 79(7) (2021) 536-544.
    e. D.G. Foushee, J.D. Moriarty, D.M. Simpson, Effects of mandibular orthognathic treatment on mucogingival tissues, J Periodontol 56(12) (1985) 727-33.
    f. V. Gandhi, P. Singh, R. Bakshi, R. Singh, R. Sood, A.S. Brar, Analysis of Age and Gender-Related Changes in Gingival Width in Patients of Known Population: A Cross-sectional Study, J Pharm Bioallied Sci 13(Suppl 1) (2021) S436-S439.
    g. W.D. Jing, J. Jiao, L. Xu, J.X. Hou, X.T. Li, X.X. Wang, X. Xu, M.X. Mao, Periodontal soft- and hard-tissue changes after augmented corticotomy in Chinese adult patients with skeletal Angle Class III malocclusion: A non-randomized controlled trial, J Periodontol 91(11) (2020) 1419-1428.
    h. J. Kong, J. Aps, S. Naoum, R. Lee, L.A. Miranda, K. Murray, J.K. Hartsfield, M.S. Goonewardene, An evaluation of gingival phenotype and thickness as determined by indirect and direct methods, The Angle orthodontist (2023).
    i. W.H. Ng, B.T. Goh, A.A.T. Lim, M.H. Tan, Mandibular asymmetry: Is there a difference in the bone and soft tissue thickness between both sides?, Oral Surg Oral Med Oral Pathol Oral Radiol 134(6) (2022) e299-e306.

2) Same patient collective (N=3)

a. E. Kalina, M. Zadurska, E. Sobieska, B. Gorski, Relationship between periodontal status of mandibular incisors and selected cephalometric parameters : Preliminary results, J Orofac Orthop 80(3) (2019) 107-115.
b. H.P. Muller, A. Heinecke, The influence of gingival dimensions on bleeding upon probing in young adults with plaque-induced gingivitis, Clin Oral Investig 6(2) (2002) 69-74.
c. A. Pascual, L. Barallat, A. Santos, P. Levi, Jr., M. Vicario, J. Nart, K. Medina, G.E. Romanos, Comparison of Periodontal Biotypes Between Maxillary and Mandibular Anterior Teeth: A Clinical and Radiographic Study, Int J Periodontics Restorative Dent 37(4) (2017) 533-539.

3) Did not meet exclusion criteria (N=4)
a. I.P. Lin, E.H. Lai, C.L. Lai, F.Y. Su, Retrospective evaluation of gingival thickness and tissue stability after connective tissue grafting in Asian populations: 1 month to 3.5 years, J Formos Med Assoc 120(5) (2021) 1242-1248.
b. H.P. Muller, K.M. Barrieshi-Nusair, E. Kononen, Repeatability of ultrasonic determination of gingival thickness, Clin Oral Investig 11(4) (2007) 439-42.
c. P.S. Choukhe D, Ambarkar S, Lavate A, Hoshing S, Shaikh S, Evaluation of Thickness of Buccal Attached Gingiva at Common Miniscrew Insertion Sites in Orthodonitc Patients with Different Facial Types, JMSCR 09(03) (2021) 215-223.
d. C.A. Figueredo, L.H. Le, K.C. Nguyen, T.G. La, E.H.M. Lou, N.R. Kaipatur, H. Lai, M.P. Gibson, C. Flores-Mir, P.W. Major, F.T. Almeida, Ultrasound Imaging of the Periodontium Complex: A Reliability Study, International Journal of Dentistry 2023 (2023).

4) Did not meet inclusion criteria (N=7)
a. D. Kloukos, E. Kalimeri, G. Koukos, A. Stahli, A. Sculean, C. Katsaros, Gingival thickness threshold and probe visibility through soft tissue: a cross-sectional study, Clin Oral Investig (2022).
b. E. Kalina, M. Zadurska, B. Gorski, Postorthodontic lower incisor and canine inclination and labial gingival recession in adult patients : A prospective study, J Orofac Orthop 82(4) (2021) 246-256.
c. S. Kakizaki, A. Aoki, M. Tsubokawa, T. Lin, K. Mizutani, G. Koshy, A. Sadr, S. Oda, Y. Sumi, Y. Izumi, Observation and determination of periodontal tissue profile using optical coherence tomography, J Periodontal Res 53(2) (2018) 188-199.
d. J. Rossell, A. Puigdollers, M. Girabent-Farres, A simple method for measuring thickness of gingiva and labial bone of mandibular incisors, Quintessence Int 46(3) (2015) 265-71.
e. A. Zimbran, S. Dudea, D. Dudea, Evaluation of periodontal tissues using 40MHz ultrasonography. preliminary report, Med Ultrason 15(1) (2013) 6-9.
f. J. Kong, J.K. Hartsfield, J. Aps, S. Naoum, R. Lee, L.A. Miranda, M.S. Goonewardene, Effect of craniofacial morphology on gingival parameters of mandibular incisors, Angle Orthod 93(5) (2023) 545-551.
g. A.S. Albughaylil, A.J. Sayed, M.A. Alsoli, M.M. Almutairi, S.F. Mohsin, S.S. Shaikh, K.A. Alsaykhan, I.A. Albulayhid, Gingival biotypes and its relation to biologic width, alveolar bone thickness, dehiscence and fenestration in mandibular anterior region: A CBCT analysis study, Journal of Pharmacy and Bioallied Sciences 15(5) (2023) S367-S371.

5) No assessment of gingiva thickness in aesthetic zone (N=7)
a. G.D. Goaslind, P.B. Robertson, C.J. Mahan, W.W. Morrison, J.V. Olson, Thickness of facial gingiva, J Periodontol 48(12) (1977) 768-71.

b. M. Sun, W. Yao, Y.Q. Deng, J. Cao, H. Meng, Measurements of buccal gingival and alveolar crest thicknesses of premolars using a noninvasive method, Med Ultrason 22(4) (2020) 409-414.
c. X.Y. Wang, Y.W. Shi, S. Zhou, Y.H. Chen, S. Yan, Evaluation of the accuracy of cone-beam computed tomography for measuring intraoral soft tissue thickness, International journal of clinical and experimental medicine 15(1) (2022) 37-44.
d. R. Parmar, V. Reddy, S.K. Reddy, D. Reddy, Determination of soft tissue thickness at orthodontic miniscrew placement sites using ultrasonography for customizing screw selection, Am J Orthod Dentofacial Orthop 150(4) (2016) 651-658.
e. L.A. Solanki, R.K. Jain, Evaluation of Thickness of Attached Gingiva at Common Mini Implant Insertion Sites, International Journal of Pharmaceutical Research 12(03) (2020) 2631-2639.
f. O. Alkan, Y. Kaya, The thickness of posterior buccal attached gingiva at common miniscrew insertion sites in subjects with different facial types, Am J Orthod Dentofacial Orthop 156(6) (2019) 800-807.
g. A. Zaragoza Ballester, Á. Ferrando Cascales, J.M. Barrera Mora, I. Friedlander, R. Agustín-Panadero, R. Ferrando Cascales, Soft- and Hard-Tissue Thicknesses in Patients with Different Vertical Facial Patterns and the Transverse Deficiencies, An Integrated CBCT-3D Digital Model Analysis, Journal of Clinical Medicine 12(4) (2023).

Table S2: Risk of Bias

Assessment of risk of bias according to RoBANS (Risk of Bias Assessment of Non-Randomized Studies):

Study	Selection of participants	Confounding variable	Measurement of exposure	Blinding of outcome assessments	Incomplete outcome data	Selective outcome reporting
Anand et al. (2022)	Low	Low	Low	Low	Low	Low
La Rocca et al. (2012)	Low	High [1, 2, 3]	Low	Low	Low	Low
Lee et al. (2017)	Low	Low	Low	Low	Low	Low
Park et al. (2017)	Low	Unclear [1]	Low	Low	Low	Low
Ranga et al. (2015)	Unclear[4]	Unclear[3]	Low	Low	Low	Low
Vandana et al. (2005)	Unclear[4]	Unclear[3]	Low	Low	Low	Low
Eger et al. (1996)	Unclear[4]	Unclear[3]	Low	Low	Low	Low
Kydd et al. (1971)	High[5]	High[1,2,3]	High[6]	Low	Low	Low
Müller et al. (2000)	Low	Low	Low	Low	Low	Low
Shao et al. (2018)	Low	Low	Low	Low	Low	Low
Han et al. (2022)	Unclear[4]	Unclear[1]	Low	Low	Low	Low

[1] no controlling for patients` sex

[2] no controlling for patients` age

[3] no assessment of history of orthodontic treatment

[4] no time frame of recruiting process reported

[5] no inclusion/exclusion criteria

[6] use of experimental measurement techniques

Figure S1: Results of subgroup analysis

Study or Subgroup	Mandible Mean	Mandible SD	Mandible Total	Maxilla Mean	Maxilla SD	Maxilla Total	Weight	Mean difference IV, Random, 95% CI
**1.2.1 supracrestal**								
Anand et al. 11-21-31-41	1.6	0.5	71	1.3	0.4	71	3.6%	0.30 [0.15 , 0.45]
Anand et al. 12-22-32-42	1.7	0.3	71	1.2	0.4	71	3.7%	0.50 [0.38 , 0.62]
Anand et al. 13-23-33-44	0.9	0.3	71	0.7	0.3	71	3.8%	0.20 [0.10 , 0.30]
Eger et al. 11-21-31-41	0.87	0.33	84	1.28	0.4	84	3.8%	-0.41 [-0.52 , -0.30]
Eger et al. 12-22-32-42	0.91	0.35	84	1.14	0.39	84	3.8%	-0.23 [-0.34 , -0.12]
Eger et al. 13-23-33-43	0.83	0.29	84	0.89	0.34	84	3.8%	-0.06 [-0.16 , 0.04]
Han et al.	0.93	0.21	94	1.29	0.33	89	3.9%	-0.36 [-0.44 , -0.28]
La Rocca et al. 11-21-31-41	0.89	0.45	30	1.23	0.79	30	2.5%	-0.34 [-0.67 , -0.01]
La Rocca et al. 12-22-32-42	0.89	0.47	30	1.05	0.5	30	3.0%	-0.16 [-0.41 , 0.09]
La Rocca et al. 13-23-33-43	1.01	0.85	30	1.01	0.54	30	2.4%	0.00 [-0.36 , 0.36]
Müller et al. 11-21-31-41	0.65	0.14	80	1	0.3	80	3.9%	-0.35 [-0.42 , -0.28]
Müller et al. 12-22-32-42	0.71	0.17	80	0.86	0.33	79	3.9%	-0.15 [-0.23 , -0.07]
Müller et al. 13-23-33-43	0.66	0.15	80	0.7	0.15	80	4.0%	-0.04 [-0.09 , 0.01]
Park et al. 11-21-31-41	0.79	0.3	95	0.96	0.34	59	3.8%	-0.17 [-0.28 , -0.06]
Shao et al. 11-21-31-41	0.89	0.23	62	1.36	0.24	62	3.9%	-0.47 [-0.55 , -0.39]
Shao et al. 12-22-32-42	0.84	0.24	62	1.16	0.19	62	3.9%	-0.32 [-0.40 , -0.24]
Shao et al. 13-23-33-43	0.83	0.2	62	1.1	0.23	62	3.9%	-0.27 [-0.35 , -0.19]
**Subtotal (95% CI)**			**1170**			**1128**	**61.6%**	**-0.14 [-0.25 , -0.02]**
Heterogeneity: Tau² = 0.05; Chi² = 392.99, df = 16 (P < 0.00001); I² = 96%								
Test for overall effect: Z = 2.37 (P = 0.02)								
**1.2.2 subcrestal**								
Han et al.*	0.76	0.18	94	0.87	0.3	89	3.9%	-0.11 [-0.18 , -0.04]
Kydd et al. 13-43*	0.69	0.36	16	1.1	0.48	16	2.7%	-0.41 [-0.70 , -0.12]
Kydd et al. 23-33*	0.5	0.32	16	0.77	0.36	16	3.1%	-0.27 [-0.51 , -0.03]
La Rocca et al. 11-21-31-41*	1.03	0.47	30	1.28	0.55	30	2.9%	-0.25 [-0.51 , 0.01]
La Rocca et al. 12-22-32-42*	0.95	0.4	30	1.12	0.46	30	3.2%	-0.17 [-0.39 , 0.05]
La Rocca et al. 13-23-33-43*	1	0.49	30	1.01	0.44	30	3.1%	-0.01 [-0.25 , 0.23]
Lee et al. 11-21-31-41*	1.01	0.31	92	1.55	0.3	93	3.9%	-0.54 [-0.63 , -0.45]
Lee et al. 12-22-32-42*	1.04	0.28	95	1.3	0.3	96	3.9%	-0.26 [-0.34 , -0.18]
Lee et al. 13-23-33-43*	1.02	0.27	95	1.26	0.4	99	3.8%	-0.24 [-0.34 , -0.14]
Ranga et al.*	1.16	0.21	240	1.1	0.15	240	4.0%	0.06 [0.03 , 0.09]
Vandana et al.*	1.07	0.34	192	1	0.32	192	3.9%	0.07 [0.00 , 0.14]
**Subtotal (95% CI)**			**930**			**931**	**38.4%**	**-0.19 [-0.32 , -0.05]**
Heterogeneity: Tau² = 0.05; Chi² = 230.92, df = 10 (P < 0.00001); I² = 96%								
Test for overall effect: Z = 2.71 (P = 0.007)								
**Total (95% CI)**			**2100**			**2059**	**100.0%**	**-0.16 [-0.24 , -0.07]**
Heterogeneity: Tau² = 0.05; Chi² = 669.62, df = 27 (P < 0.00001); I² = 96%								
Test for overall effect: Z = 3.60 (P = 0.0003)								
Test for subgroup differences: Chi² = 0.28, df = 1 (P = 0.60), I² = 0%								

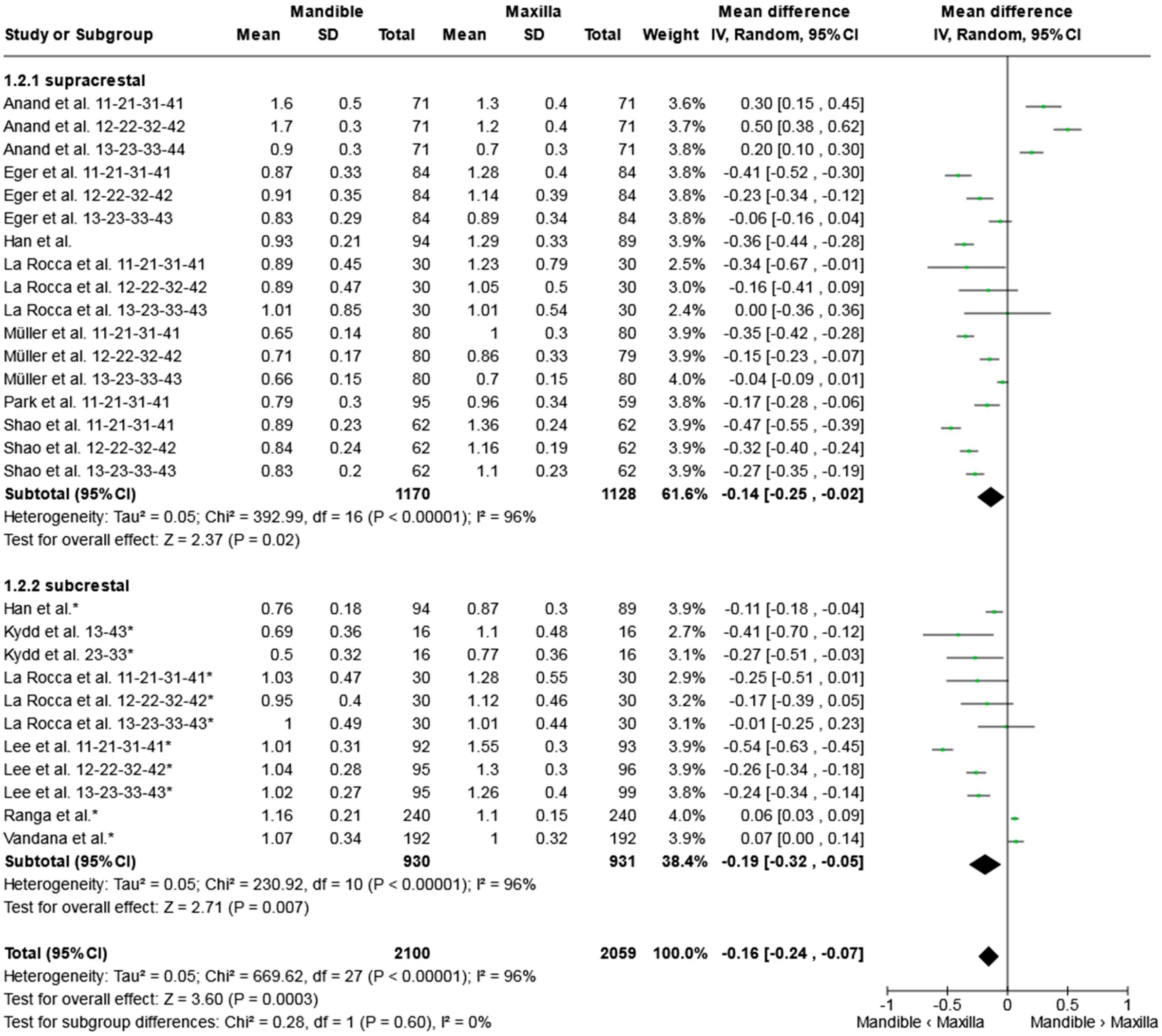

Study or Subgroup	Mandible Mean	Mandible SD	Mandible Total	Maxilla Mean	Maxilla SD	Maxilla Total	Weight	Mean difference IV, Random, 95% CI
**1.2.1 CBCT**								
Han et al.	0.93	0.21	94	1.29	0.33	89	3.9%	-0.36 [-0.44 , -0.28]
Han et al.*	0.76	0.18	94	0.87	0.3	89	3.9%	-0.11 [-0.18 , -0.04]
**Subtotal (95% CI)**			**188**			**178**	**7.8%**	**-0.23 [-0.48 , 0.01]**
Heterogeneity: Tau² = 0.03; Chi² = 20.50, df = 1 (P < 0.00001); I² = 95%								
Test for overall effect: Z = 1.87 (P = 0.06)								
**1.2.2 Ultrasound**								
Eger et al. 11-21-31-41	0.87	0.33	84	1.28	0.4	84	3.8%	-0.41 [-0.52 , -0.30]
Eger et al. 12-22-32-42	0.91	0.35	84	1.14	0.39	84	3.8%	-0.23 [-0.34 , -0.12]
Eger et al. 13-23-33-43	0.83	0.29	84	0.89	0.34	84	3.8%	-0.06 [-0.16 , 0.04]
Kydd et al. 13-43*	0.69	0.36	16	1.1	0.48	16	2.7%	-0.41 [-0.70 , -0.12]
Kydd et al. 23-33*	0.5	0.32	16	0.77	0.36	16	3.1%	-0.27 [-0.51 , -0.03]
Müller et al. 11-21-31-41	0.65	0.14	80	1	0.3	80	3.9%	-0.35 [-0.42 , -0.28]
Müller et al. 12-22-32-42	0.71	0.17	80	0.86	0.33	79	3.9%	-0.15 [-0.23 , -0.07]
Müller et al. 13-23-33-43	0.66	0.15	80	0.7	0.15	80	4.0%	-0.04 [-0.09 , 0.01]
**Subtotal (95% CI)**			**524**			**523**	**28.9%**	**-0.23 [-0.34 , -0.11]**
Heterogeneity: Tau² = 0.02; Chi² = 80.85, df = 7 (P < 0.00001); I² = 91%								
Test for overall effect: Z = 3.83 (P = 0.0001)								
**1.2.3 Transgingival**								
Anand et al. 11-21-31-41	1.6	0.5	71	1.3	0.4	71	3.6%	0.30 [0.15 , 0.45]
Anand et al. 12-22-32-42	1.7	0.3	71	1.2	0.4	71	3.7%	0.50 [0.38 , 0.62]
Anand et al. 13-23-33-44	0.9	0.3	71	0.7	0.3	71	3.8%	0.20 [0.10 , 0.30]
La Rocca et al. 11-21-31-41	0.89	0.45	30	1.23	0.79	30	2.5%	-0.34 [-0.67 , -0.01]
La Rocca et al. 11-21-31-41*	1.03	0.47	30	1.28	0.55	30	2.9%	-0.25 [-0.51 , 0.01]
La Rocca et al. 12-22-32-42	0.89	0.47	30	1.05	0.5	30	3.0%	-0.16 [-0.41 , 0.09]
La Rocca et al. 12-22-32-42*	0.95	0.4	30	1.12	0.46	30	3.2%	-0.17 [-0.39 , 0.05]
La Rocca et al. 13-23-33-43	1.01	0.85	30	1.01	0.54	30	2.4%	0.00 [-0.36 , 0.36]
La Rocca et al. 13-23-33-43*	1	0.49	30	1.01	0.44	30	3.1%	-0.01 [-0.25 , 0.23]
Lee et al. 11-21-31-41*	1.01	0.31	92	1.55	0.3	93	3.9%	-0.54 [-0.63 , -0.45]
Lee et al. 12-22-32-42*	1.04	0.28	95	1.3	0.3	96	3.9%	-0.26 [-0.34 , -0.18]
Lee et al. 13-23-33-43*	1.02	0.27	95	1.26	0.4	99	3.8%	-0.24 [-0.34 , -0.14]
Park et al. 11-21-31-41	0.79	0.3	95	0.96	0.34	59	3.8%	-0.17 [-0.28 , -0.06]
Ranga et al.*	1.16	0.21	240	1.1	0.15	240	4.0%	0.06 [0.03 , 0.09]
Shao et al. 11-21-31-41	0.89	0.23	62	1.36	0.24	62	3.9%	-0.47 [-0.55 , -0.39]
Shao et al. 12-22-32-42	0.84	0.24	62	1.16	0.19	62	3.9%	-0.32 [-0.40 , -0.24]
Shao et al. 13-23-33-43	0.83	0.2	62	1.1	0.23	62	3.9%	-0.27 [-0.35 , -0.19]
Vandana et al.*	1.07	0.34	192	1	0.32	192	3.9%	0.07 [0.00 , 0.14]
**Subtotal (95% CI)**			**1388**			**1358**	**63.3%**	**-0.11 [-0.24 , 0.01]**
Heterogeneity: Tau² = 0.07; Chi² = 532.18, df = 17 (P < 0.00001); I² = 97%								
Test for overall effect: Z = 1.78 (P = 0.08)								
**Total (95% CI)**			**2100**			**2059**	**100.0%**	**-0.16 [-0.24 , -0.07]**
Heterogeneity: Tau² = 0.05; Chi² = 669.62, df = 27 (P < 0.00001); I² = 96%								
Test for overall effect: Z = 3.60 (P = 0.0003)								
Test for subgroup differences: Chi² = 1.85, df = 2 (P = 0.40), I² = 0%								

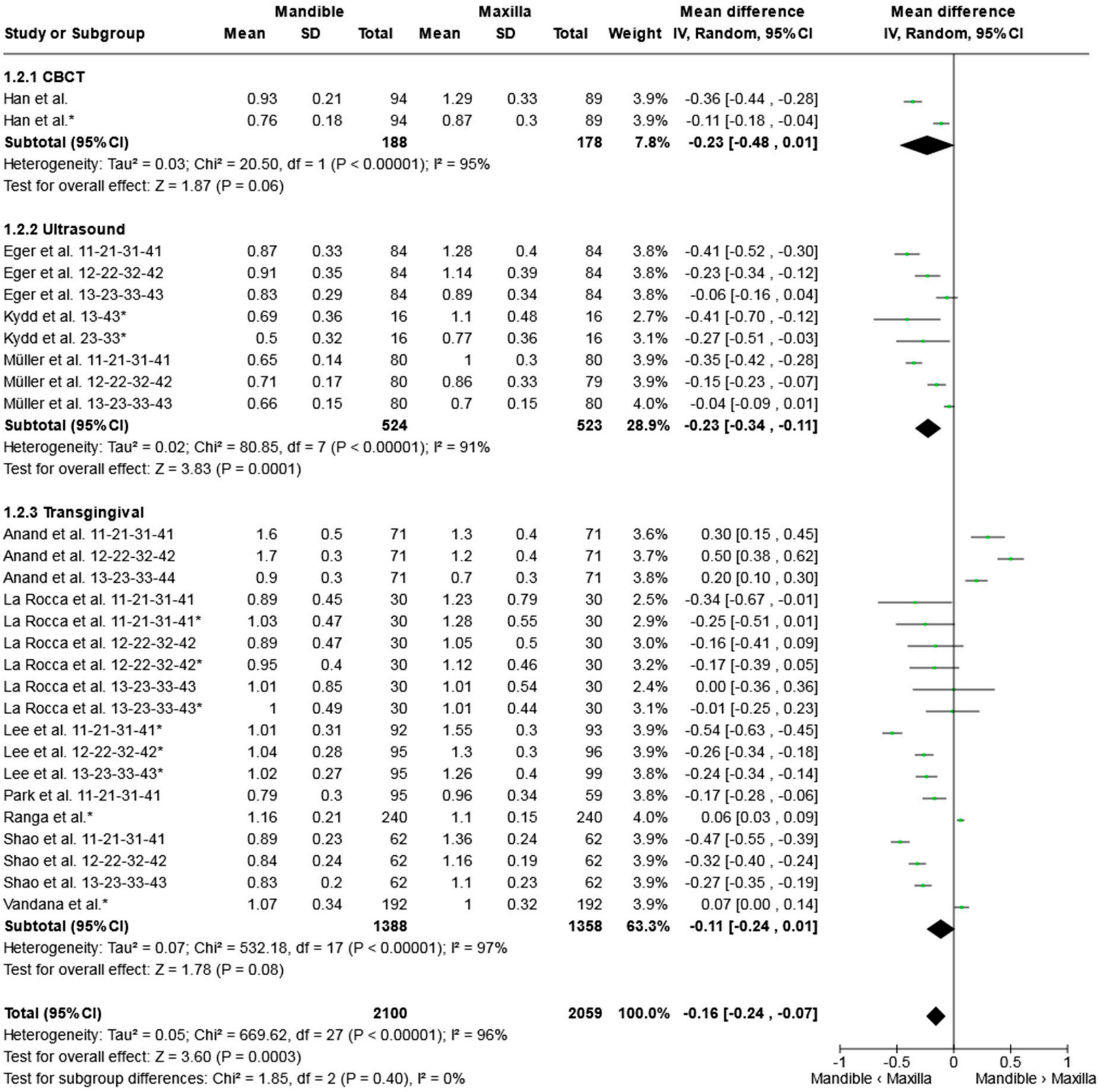

Study or Subgroup	Mandible Mean	Mandible SD	Mandible Total	Maxilla Mean	Maxilla SD	Maxilla Total	Weight	Mean difference IV, Random, 95% CI
**1.2.1 Canine**								
Anand et al. 13-23-33-44	0.9	0.3	71	0.7	0.3	71	4.5%	0.20 [0.10 , 0.30]
Eger et al. 13-23-33-43	0.83	0.29	84	0.89	0.34	84	4.5%	-0.06 [-0.16 , 0.04]
Kydd et al. 13-43*	0.69	0.36	16	1.1	0.48	16	3.3%	-0.41 [-0.70 , -0.12]
Kydd et al. 23-33*	0.5	0.32	16	0.77	0.36	16	3.7%	-0.27 [-0.51 , -0.03]
La Rocca et al. 13-23-33-43*	1	0.49	30	1.01	0.44	30	3.7%	-0.01 [-0.25 , 0.23]
Lee et al. 13-23-33-43*	1.02	0.27	95	1.26	0.4	99	4.5%	-0.24 [-0.34 , -0.14]
Müller et al. 13-23-33-43	0.66	0.15	80	0.7	0.15	80	4.7%	-0.04 [-0.09 , 0.01]
Shao et al. 13-23-33-43	0.83	0.2	62	1.1	0.23	62	4.6%	-0.27 [-0.35 , -0.19]
**Subtotal (95% CI)**			**454**			**458**	**33.5%**	**-0.12 [-0.24 , 0.00]**
Heterogeneity: Tau² = 0.02; Chi² = 76.25, df = 7 (P < 0.00001); I² = 91%								
Test for overall effect: Z = 1.95 (P = 0.05)								
**1.2.2 Incisors**								
Anand et al. 11-21-31-41	1.6	0.5	71	1.3	0.4	71	4.3%	0.30 [0.15 , 0.45]
Anand et al. 12-22-32-42	1.7	0.3	71	1.2	0.4	71	4.4%	0.50 [0.38 , 0.62]
Eger et al. 11-21-31-41	0.87	0.33	84	1.28	0.4	84	4.5%	-0.41 [-0.52 , -0.30]
Eger et al. 12-22-32-42	0.91	0.35	84	1.14	0.39	84	4.5%	-0.23 [-0.34 , -0.12]
La Rocca et al. 11-21-31-41	0.89	0.45	30	1.23	0.79	30	3.1%	-0.34 [-0.67 , -0.01]
La Rocca et al. 11-21-31-41*	1.03	0.47	30	1.28	0.55	30	3.5%	-0.25 [-0.51 , 0.01]
La Rocca et al. 12-22-32-42	0.89	0.47	30	1.05	0.5	30	3.6%	-0.16 [-0.41 , 0.09]
La Rocca et al. 12-22-32-42*	0.95	0.4	30	1.12	0.46	30	3.8%	-0.17 [-0.39 , 0.05]
La Rocca et al. 13-23-33-43	1.01	0.85	30	1.01	0.54	30	2.8%	0.00 [-0.36 , 0.36]
Lee et al. 11-21-31-41*	1.01	0.31	92	1.55	0.3	93	4.6%	-0.54 [-0.63 , -0.45]
Lee et al. 12-22-32-42*	1.04	0.28	95	1.3	0.3	96	4.6%	-0.26 [-0.34 , -0.18]
Müller et al. 11-21-31-41	0.65	0.14	80	1	0.3	80	4.6%	-0.35 [-0.42 , -0.28]
Müller et al. 12-22-32-42	0.71	0.17	80	0.86	0.33	79	4.6%	-0.15 [-0.23 , -0.07]
Park et al. 11-21-31-41	0.79	0.3	95	0.96	0.34	59	4.5%	-0.17 [-0.28 , -0.06]
Shao et al. 11-21-31-41	0.89	0.23	62	1.36	0.24	62	4.6%	-0.47 [-0.55 , -0.39]
Shao et al. 12-22-32-42	0.84	0.24	62	1.16	0.19	62	4.6%	-0.32 [-0.40 , -0.24]
**Subtotal (95% CI)**			**1026**			**991**	**66.5%**	**-0.19 [-0.32 , -0.06]**
Heterogeneity: Tau² = 0.06; Chi² = 310.79, df = 15 (P < 0.00001); I² = 95%								
Test for overall effect: Z = 2.93 (P = 0.003)								
**Total (95% CI)**			**1480**			**1449**	**100.0%**	**-0.17 [-0.27 , -0.08]**
Heterogeneity: Tau² = 0.05; Chi² = 450.08, df = 23 (P < 0.00001); I² = 95%								
Test for overall effect: Z = 3.49 (P = 0.0005)								
Test for subgroup differences: Chi² = 0.63, df = 1 (P = 0.43), I² = 0%								

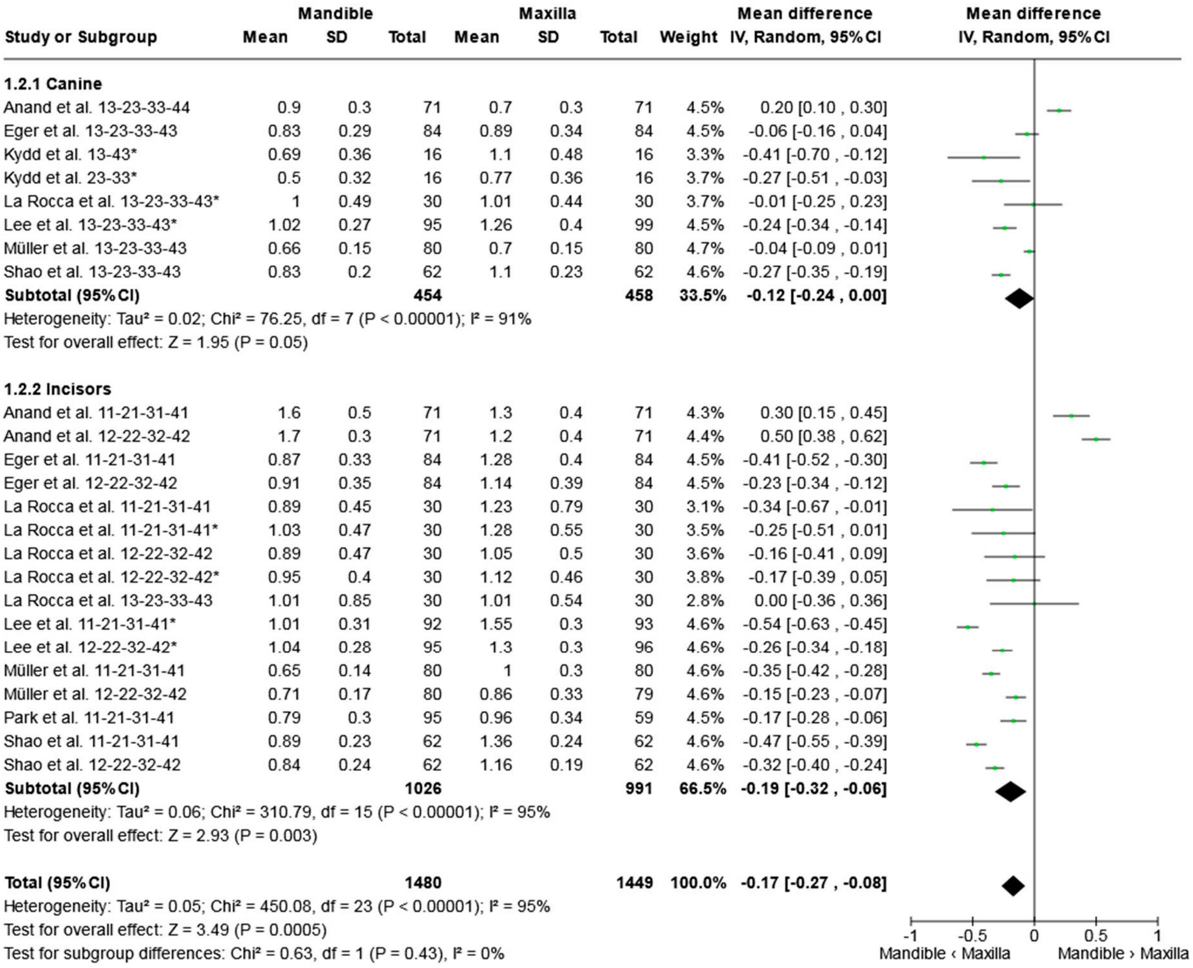

Table S3:

Summary of findings using the Grading of Recommendations Assessment, Development and Evaluation (GRADE) approach.

Difference in gingiva thickness between upper and lower jaw

Patient or population: Healthy patients with permanent dentition

Setting: University/Private clinics

Intervention: Transgingival probing, ultrasound measurement or CBCT

Outcomes: Facial gingiva thickness in mm

**Outcomes***	**WME (95% CI)** Effect in patients without signs of periodontitis	**№ of teeth (studies)**	**Certainty of the evidence (GRADE)**	**Comments**
Difference in facial gingiva thickness around anterior teeth	Mean difference of gingiva: in **maxilla** 0.16 mm **thicker** (0.07 to 0.24 mm thicker)	2100 lower teeth, 2059 upper teeth (11 studies)	⨁◯◯◯ VERY LOW [a,b,c] Due to risk of bias and indirectness	Gingiva thickness may be slightly thicker in the upper jaw. The currently available evidence shows a difference of 0.16 mm.

CI: Confidence interval; **WME:** Weighted mean effects

Explanations:

a. Starts from “low” as observational cross-sectional studies
b. Risk of bias due to deficiencies in the domain of selection
c. Inconsistency of results due to large Heterogeneity
